# The Clinical Significance and Molecular Features of the Spatial Tumor Shapes in Breast Cancers

**DOI:** 10.1371/journal.pone.0143811

**Published:** 2015-12-15

**Authors:** Hyeong-Gon Moon, Namshin Kim, Seongmun Jeong, Minju Lee, HyunHye Moon, Jongjin Kim, Tae-Kyung Yoo, Han-Byoel Lee, Jisun Kim, Dong-Young Noh, Wonshik Han

**Affiliations:** 1 Department of Surgery, Seoul National University College of Medicine, Seoul, Korea; 2 Laboratory of Breast Cancer Biology, Cancer Research Institute, Seoul National University College of Medicine, Seoul, Korea; 3 Genome Medicine Institute, Seoul National University College of Medicine, Seoul, Korea; 4 Epigenomics Research Center, Genome Institute, Korea Research Institute of Bioscience and Biotechnology, Daejeon, Korea; Taipei Medical University, TAIWAN

## Abstract

Each breast cancer has its unique spatial shape, but the clinical importance and the underlying mechanism for the three-dimensional tumor shapes are mostly unknown. We collected the data on the three-dimensional tumor size and tumor volume data of invasive breast cancers from 2,250 patients who underwent surgery between Jan 2000 and Jul 2007. The degree of tumor eccentricity was estimated by using the difference between the spheroid tumor volume and ellipsoid tumor volume (spheroid-ellipsoid discrepancy, SED). In 41 patients, transcriptome and exome sequencing data obtained. Estimation of more accurate tumor burden by calculating ellipsoid tumor volumes did not improve the outcome prediction when compared to the traditional longest diameter measurement. However, the spatial tumor eccentricity, which was measured by SED, showed significant variation between the molecular subtypes of breast cancer. Additionally, the degree of tumor eccentricity was associated with well-known prognostic factors of breast cancer such as tumor size and lymph node metastasis. Transcriptome data from 41 patients showed significant association between MMP13 and spatial tumor shapes. Network analysis and analysis of TCGA gene expression data suggest that MMP13 is regulated by ERBB2 and S100A7A. The present study validates the usefulness of the current tumor size method in determining tumor stages. Furthermore, we show that the tumors with high eccentricity are more likely to have aggressive tumor characteristics. Genes involved in the extracellular matrix remodeling can be candidate regulators of the spatial tumor shapes in breast cancer.

## Introduction

The amount of cancer cell accumulation that is reflected by the tumor’s spatial shape is the result of constant interaction between the proliferating cancer cells and their microenvironment. Along their spatial growth, the cells of solid cancers initiate the process of invasion and metastasis that can ultimately lead to fatal distant diseases. The degree of tumor cell accumulation in the primary organ is often measured by the longest diameter, an integral component of the widely used TNM staging system [1,2]. The largest tumor diameter is regarded to represent the risk of cancer metastasis and the probability of distant recurrences. However, the degree of cancer cell accumulation can be poorly determined when the tumor size is solely assessed by the uni-dimensional diameter since each human tumor has a unique three-dimensional shape. Accordingly, researchers have proposed better prognostic models based on the tumor volume measurement rather than using single diameter for various types of cancers [3,4]. The optimal method of measuring tumor burden in the primary organ remains to be tested.

Another issue that should be addressed with regard to the spatial tumor growth is the clinical implications and the underlying mechanisms for the inter-tumoral variations of the spatial tumor shapes. It is largely unknown how each tumor shape its spatial contour and what are the underlying differences in molecular characteristics. Recent studies are now beginning to elucidate the molecular characteristics of this spatial tumor growth. For example, colorectal tumors that show laterally spreading patterns show unique gene expression features including β-catenin, type IV collagen, and aPKC [5]. Mathematical modeling of the spatial tumor growth has been often used to explain the process of longitudinal tumor growth [6–8]. Although the modeling approach can reveal many novel aspects of tumor growth, the approach is limited by the difficulties in incorporating other clinical characteristics of tumors.

In this study, we aimed to explore the usefulness of the tumor volume measurement in predicting outcomes of the breast cancer patients. Additionally, we investigated the inter-tumor variations of eccentricity in three-dimensional tumor shapes and the association of this eccentricity with known important prognostic factors in breast cancer. Finally, in a small cohort of breast cancer patients, we explored the relationship between the spatial tumor shape and molecular characteristics of tumors.

## Materials and Methods

### Patients and database

The use of the clinical and pathologic data from breast cancer patients for this study was approved by the institutional IRB of Seoul National University Hospital. The written informed consents were obtained prior to the tissue collection for breast cancer tissue repository (IRB No 1405-088-580). For the retrospective analysis, the patients record and identity were anonymized and de-identified prior to analysis by approved researchers (IRB No 1504-057-664). All procedures were done in accordance with the Declaration of Helsinki.

The demographic, clinical, and pathologic information of the studied patients were obtained from the Seoul National University Hospital Breast Care Center Database. The detailed information of the database has been described previously [9]. We retrieved data of all breast cancer patients who underwent breast cancer surgery between Jan 2000 and Jul 2007. Exclusion criteria were patients with multifocal or multi centric tumors; patients who received preoperative systemic treatment, patients who underwent excisional biopsy for the diagnosis of cancer, patients with tumors larger than 10cm, patients with no available three-dimensional tumor size measurement, and patients without immunohistochemistry subtype information. Three-dimensional tumor diameters were measured by the pathologists at the time of pathologic diagnosis.

### Tumor volume measurements and spheroid-ellipsoid discrepancy (SED)

We calculated four different types of tumor volume (spheric, prolate, oblate, and ellipsoid) for each tumor by using three dimensional pathologic tumor sizes. The equations used to calculate each tumor volume were spherical tumor volume = 4/3π(a/2)^3^; oblate tumor volume = 4/3π(a/2)^2^(b/2); prolate tumor volume = 4/3π(a/2)(c/2)^2^; and ellipsoid tumor volume = 4/3π(a/2)(b/2)(c/2). The variable a, b, and c represents the largest, second largest, and the smallest diameter, respectively [10].

SED was defined as the proportional volume discrepancy between the STV and ETV of each tumor (SED = (STV-ETV)/STV). SED value increases as the tumors have more ellipsoid spatial shapes. For example, If a tumor has identical three-dimensional diameters, the SED of the tumor would be zero.

### Transcriptome and exome profiles associated with SED

The results of transcriptome and exome sequencing of 120 breast cancer tissues, which was approved by the Seoul National University Hospital IRB (IRB No 1109-007-376), has previously been reported [11]. In this study, the data of 41 breast tumors with available three- dimensional tumor diameters were analyzed. Breast cancer tissues were collected at the curative surgery for patients who gave informed consents.

Briefly, total RNA was obtained from archived tumor tissues and cDNA library was constructed with the TruSeq RNA kit. The kit protocol included polyA-selected RNA extraction, RNA fragmentation, random-hexamer-primed reverse transcription, and 101 nucleotide paired-end sequencing, which was performed on an Illumina HiSeq2000.

We had checked quality of reads by FastQC v0.11.3 and removed 5 bp at both fragment ends by NGSQCToolkit v2.3.3. We had used in-house custom script to determine fragment sizes and standard deviations after all the paired-end reads were mapped onto NCBI RefSeq transcriptome by BWA v0.7.10. Average fragment size is 195 bp and average standard deviation is 68. Tuxedo protocol (bowtie v2.2.4 and tophat v2.0.13) was used to map the reads onto hg19 human reference genome with refGene transcriptome downloaded from UCSC genome browser. We used HTSeq v0.6.1 to extract reads counts for each gene and in-house R scripts to assess the correlation and linear regression for gene expression and SED values. We have used normalized FPKM values from all RNA-Seq data in order to find positively and negatively correlated genes with SED values. Pearson correlation and p-Value were calculation by cor.test module in R package. We have chosen final candidate genes by selecting p-Value < 0.01, means > 10, and CV > 1. For pathway analysis, we have generated connection network diagrams by using Pathway Studio Web [12].

For whole exome sequencing, genomic DNA was extracted from tumor tissues and blood samples using a QIAamp DNA Mini Kit (Qiagen, Valencia, CA, USA). DNA integrity was verified by electrophoresis on 0.8% agarose gels. The quality and quantity of the DNA were measured using a NanoDrop Spectrophotometer and Quant-iT PicoGreen dsDNA Assay Kit (Thermo Fisher Scientific, Waltham, MA, USA), respectively. An amplicon library was generated using SureSelectXT whole exome v4.0 (Agilent Technologies, Santa Clara, CA, USA) and the whole-exome sequencing (WES) was performed with an Illumina HiSeq 2500 system (Illumina, Inc., San Diego, CA, USA). Among, the 41 patients, two showed poor quality in exome sequencing data, and the final exome analysis was done in 39 patients. Absolute copy numbers based on copy-number variations of samples are inferred by absCN-seq [13], and clonality analysis is performed by sciClone [14]. Somatic mutations are calculated by VarScan v2.3.6 and variants are annotated by in-house annotation pipeline.

### Quantitative real time-PCR (qRT-PCR)

Quantitative real time-PCR was performed in 96 well PCR plate (Thermo scientific) containing the SYBR green Master Mix (Applied Biosystems), distilled water, 10 ng of cDNA templates from breast cancer tissues and 200 nM of *MMP13* forward and reverse primers. Specific primer sequences are 5’-CTTGACCACTCCAAGGACCC-3’ (forward), 5’-CCTCGGAGACTGGTAATGGC-3’ (reverse). qRT-PCR analysis was performed with the 7300 Real Time PCR System (Applied Biosystems) using the following conditions: 95°C for 10 min, followed by 40 cycles of 95°C for 15 sec and 60°C for one min. The results were normalized to the housekeeping gene, β-actin, and the cycle threshold (Ct) values were analyzed. These experiments were repeated three times.

### Statistical analysis

Patients were classified into four groups according to their expression status of hormonal receptor (HR) status and HER2 amplification status. HR status was defined as positive when the tumor showed positive expression of either ER or PR. The ER and PR positivity were defined by the cut-off of 10% and HER2-positive tumors were either strong positive on immunochemical staining or gene amplification on FISH. The characteristics were compared by using the χ2 test and Student’s t-test. Univariate survival analysis was carried out using the Kaplan-Meier method, and log-rank tests were employed for comparison of survival curves. Multivariate analyses were conducted using Cox’s proportional hazard regression model. The relationship between MMP13 gene expression and molecular subtypes of breast cancer was assessed by using RNA Seq data downloaded from cbioportal (http://www.cbioportal.org) [15].

## Results

### Various types of tumor volume (TV) measurements and survival prediction

**Fig 1:**
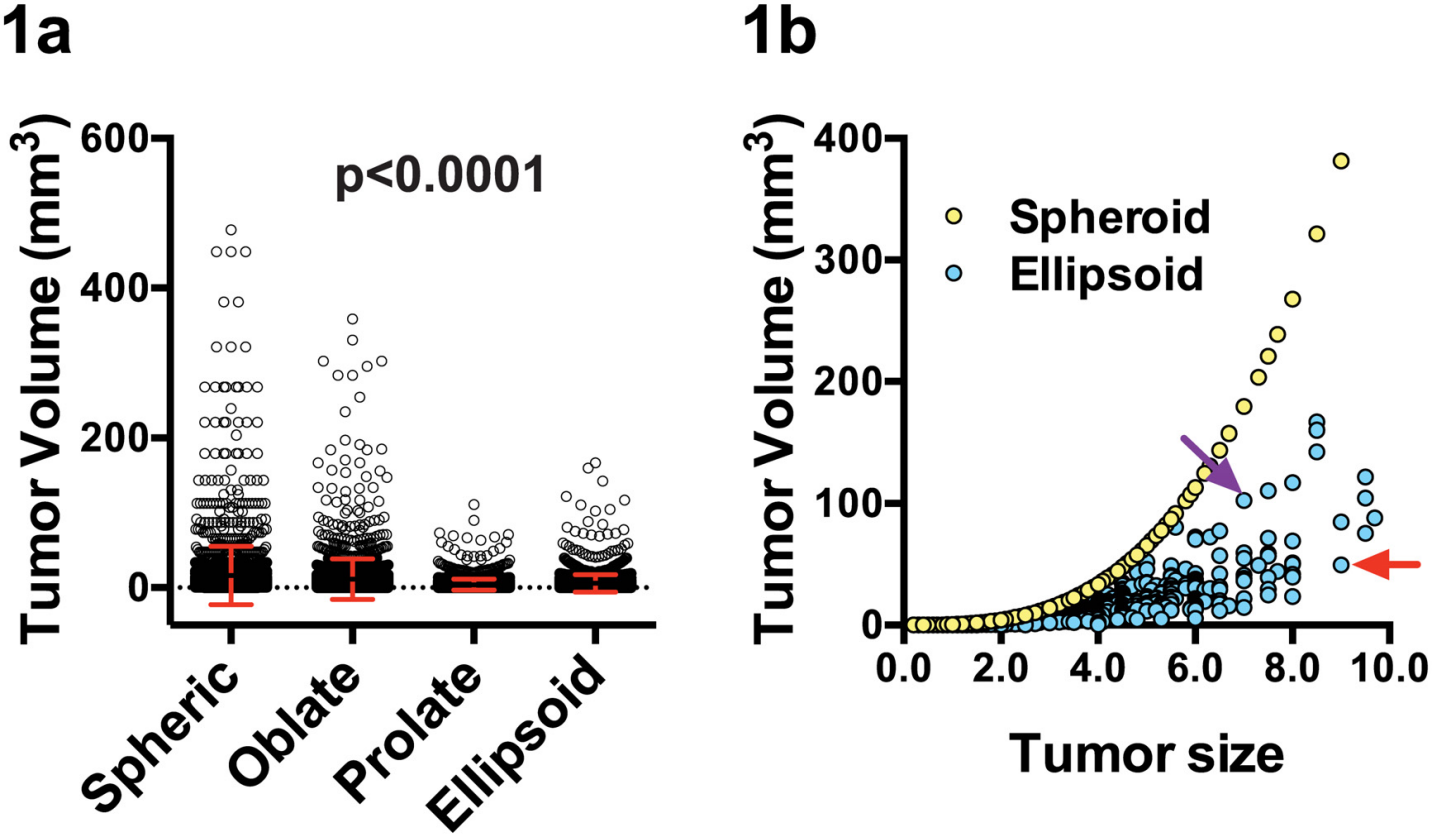
Various types of tumor volume measurements. Comparison of various tumor volume measurement methods for 2,250 primary breast tumors (1a). Spheroid tumor volumes and ellipsoid tumor volumes for 2,250 tumors according to the largest tumor size (1b). The red and purple arrows indicate the cases with discordant ellipsoid volume estimation and tumor sizes. Although the tumor with purple arrow has smaller tumor diameter than the tumor with red arrow, it has higher tumor volume with the ellipsoid TV measurement.

We calculated various types of TV measurement in 2,250 breast cancer patients who underwent surgery between Jan 2000 and Jul 2007 based on their three-dimensional diameters measured during the pathologic examinations. As shown in Fig 1a, there was a significant difference between spheroid, oblate, prolate, and ellipsoid TV measurements. The median TV was 5.57 (±39.9) cm3 for spheroid TV measurement and 2.54 (±11.5) cm3 for ellipsoid TV measurement, respectively. In some tumors, the types of TV measurement resulted in change in the ranks of tumor size as shown in the Fig 1b.

**Fig 2:**
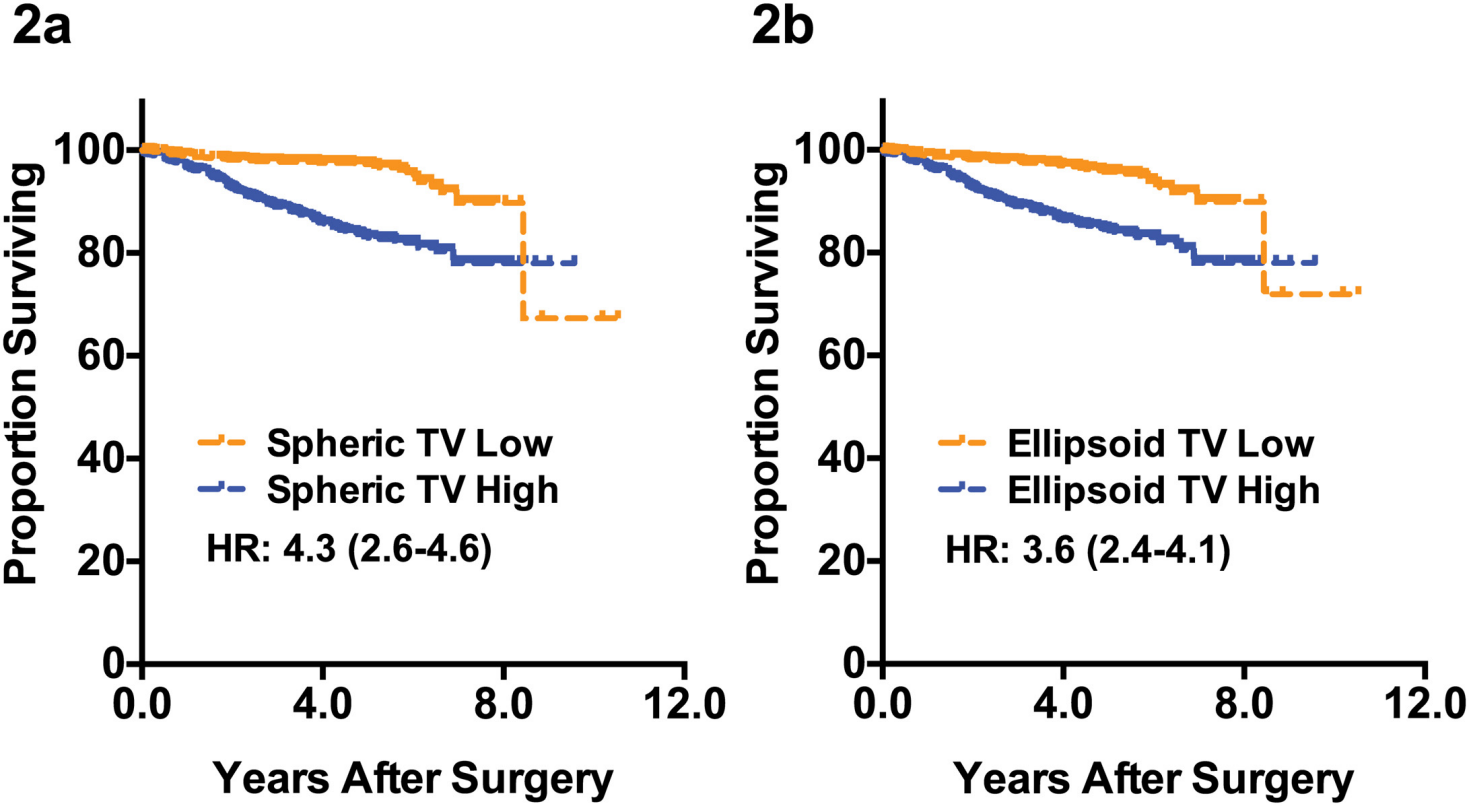
Distant metastasis-free survival according to the tumor volumes. Comparison of the prognosis predicting accuracy of the spheroid tumor volume measurement (2a) and ellipsoid tumor measurement (2b) are shown. HR: hazard ratio estimated by univariate Cox regression analysis, TV: tumor volume.

Ellipsoid TV can reflect the true tumor volume more accurately compared to spheroid TV since it takes account of all three-dimensional diameters. We analyzed whether this potentially improved TV estimation can improve the prediction of recurrence in breast cancer patients. As shown in the Fig 2, classifying patients according to the ellipsoid tumor volume did not improve the prognosis prediction. Our data suggest that improved tumor volume estimation does not lead to better survival prediction.

### The association between spatial tumor shape, SED, and molecular subtypes of breast cancer

**Fig 3:**
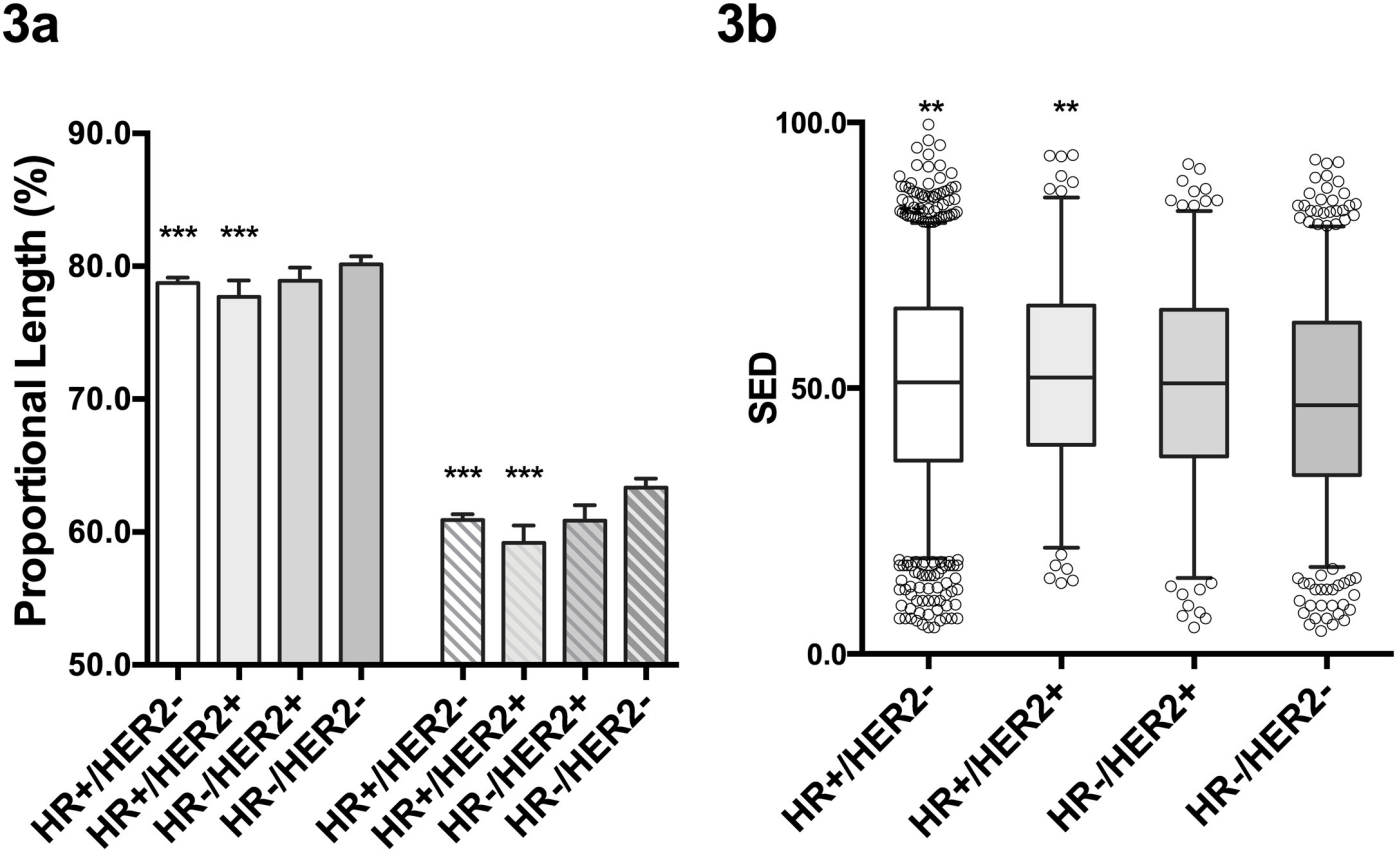
Three-dimensional tumor diameters according to the molecular subtypes. The relative lengths of b (2nd largest pathologic diameter) and c (3rd largest pathologic diameter) according to the molecular subtypes of breast cancer are shown in 3a. The bars represent the relative lengths of the b (solid bars) and c (shaded bars) in comparison to the largest diameter of the tumors (a). *** P<0.001 compared to HR-/HER2-. The distribution of the SED (spheroid-ellipsoid discrepancy) according to the molecular subtypes of breast cancer is shown in 3b. Bars represent the 5–95 percentiles. ** P<0.01 when compared to HR-/HER2- tumors. HR: hormonal receptor, HER2: HER2 overexpression.

We assessed the effect of breast cancer molecular subtypes on the spatial tumor shapes. First, we analyzed the relative lengths of the diameter b (b/a) and c (c/a) of tumors in different molecular subtypes as defined by the HR and HER2 expression status. As shown in Fig 3a, the relative lengths of b and c were significantly shorter in HR+ tumors when compared to those of the HR-/HER2- tumors. To measure the tumor eccentricity quantitatively, we calculated the spheroid-ellipsoid discrepancy (SED) for all studied tumors. The SED ranges from 0 to 1, and the SED would be 0 for the tumors that are completely spheroid. As expected, the HR-/HER2- tumors had significantly lower SED when compared to HR+ tumors (Fig 3b). In overall, the triple negative breast cancers, which are HR-/HER2- tumors, had most spheroid shape compared to other subtypes.

### Prognostic significance of the tumor eccentricity

**Fig 4:**
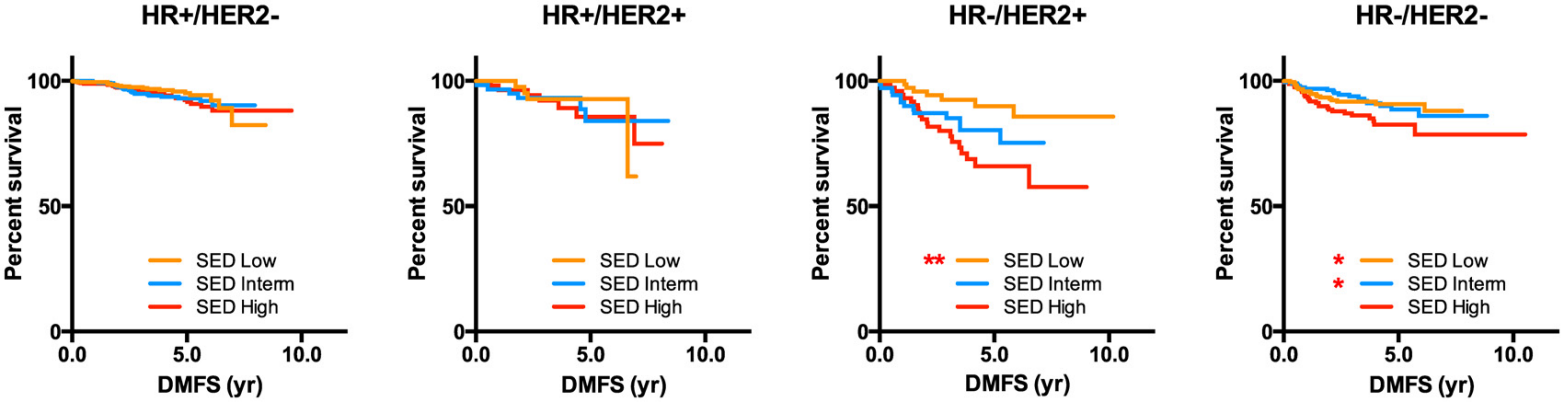
Kaplan-Meier survival curve according to tumor eccentricity. **: P<0.01, *:P<0.05, The p values are derived from the log-rank test compared to the SED High group. SED: spheroid-ellipsoid discrepancy.

The prognostic implication of the tumor eccentricity on the distant-metastasis free survival of the breast cancer patients was analyzed. There was a significant association between the degree of eccentricity and the time to distant metastasis in patients with hormone receptor negative tumors (Fig 4). Tumors with higher SED had shorter time to distant metastasis. The prognostic significance of the tumor’s SED can be attributed to the association between the SED and known poor prognostic factors such as tumor size, node metastasis, and histologic grade (Table 1). When the patients were classified according to the HR and HER2 status, the significant association between the SED and lymph node metastasis was only seen in HR-/HER2- tumors. After adjusting for other prognostic factors, the SED did not show a significant impact on distant metastasis (S1 Table). These results show that, although not an independent prognostic factor, the degree of eccentricity is associated with various clinical and pathologic prognostic factors and tumor recurrence in a subset of breast cancer patients.

**Table 1:** The tumor size and lymph node metastasis in patients classified according to the SED.

		Low SED	Middle SED	High SED	p value
All patients					
Tumor size (cm)		1.95 (±0.78)	2.33 (±1.04)	3.06 (±1.65)	<0.001
LN metastasis					
No		487 (64.9%)	428 (57.1%)	402 (53.6%)	<0.001
Yes		263 (35.1%)	321 (42.9%)	348 (46.4%)	
HR+/HER2-					
Tumor size (cm±SD)		1.78 (±0.71)	2.16 (±0.85)	2.80 (±1.53)	<0.001
LN metastasis					
No		261 (62.4%)	243 (57.3%)	248 (54.5%)	<0.057
Yes		157 (37.6%)	181 (42.7%)	207 (45.5%)	
HR+/HER2+					
Tumor size (cm±SD)		2.17 (±0.87)	2.26 (±1.27)	3.23 (±1.68)	<0.001
LN metastasis					
No		24 (54.5%)	32 (54.2%)	26 (47.3%)	0.696
Yes		20 (45.5%)	27 (45.8%)	29 (52.7%)	
HR-/HER2+					
Tumor size (cm±SD)		2.43 (±0.92)	2.75 (±1.26)	3.73 (±1.87)	<0.001
LN metastasis					
No		43 (58.9%)	39 (55.7%)	35 (47.9%)	0.393
Yes		30 (41.1%)	31 (44.3%)	38 (52.1%)	
HR-/HER2-					
Tumor size (cm±SD)		2.07 (±0.75)	2.53 (±1.15)	3.39 (±1.67)	<0.001
LN metastasis					
No		158 (73.8%)	111 (57.5%)	92 (56.4%)	<0.001
Yes		56 (26.2%)	82 (42.5%)	71 (43.6%)	

### Gene expression profiles, somatic mutations, and clonality

To investigate the gene expression profiles underlying the determination of breast cancer eccentricity, we obtained the transcriptome data generated by a paired-end massively parallel RNA sequencing of 41 breast tumors, which was a part of the breast cancer RNA sequencing project [11].

**Fig 5:**
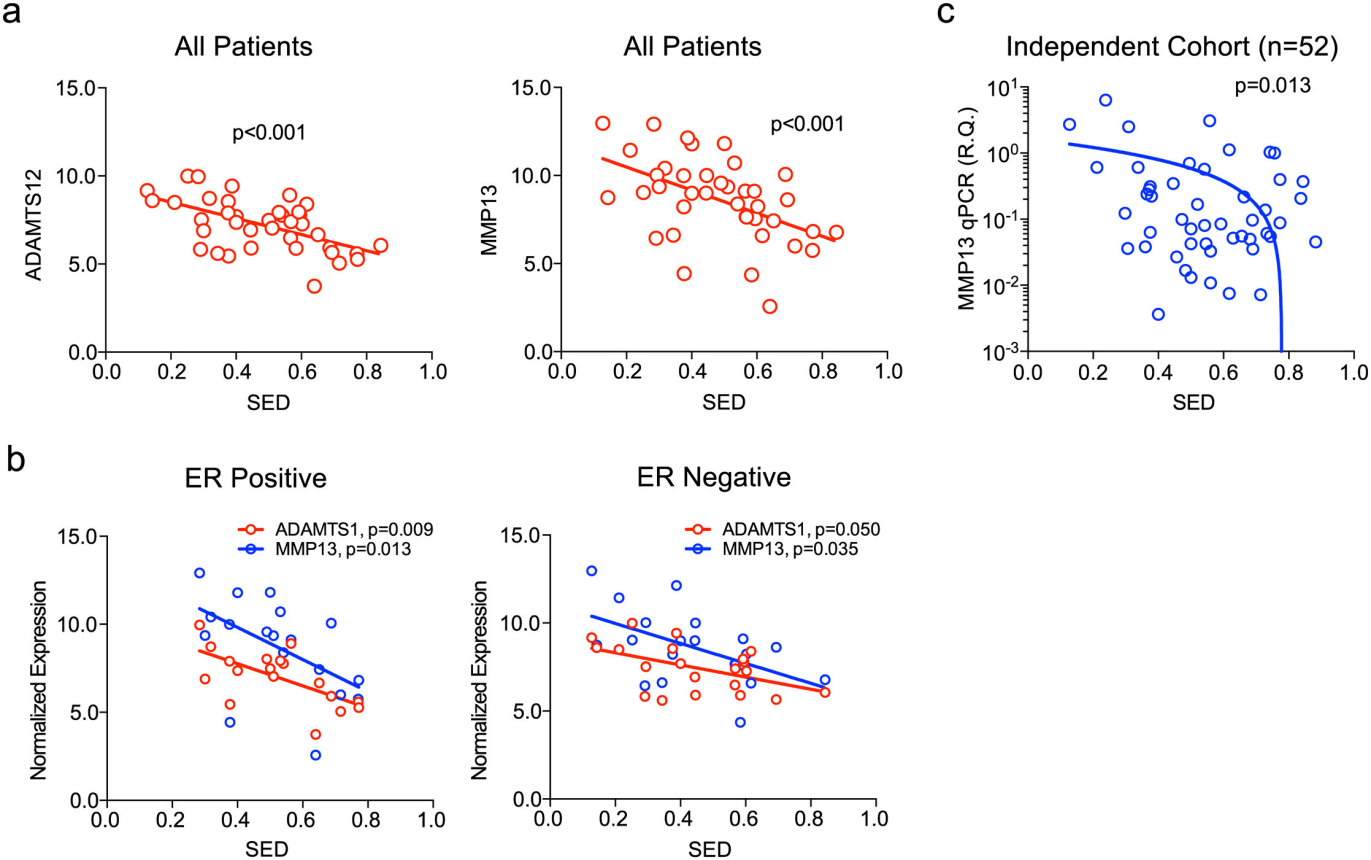
Gene expression profiles associated with breast cancer’s spatial growth measured by SED (spheroid-ellipsoid discrepancy). The scatter plots for MMP13 and ADAMTS12 are shown in (a) and the correlation was stratified according to the hormonal receptor status (b). The results of the qRT-PCR against MMP13 and the SED are shown in Fig 5c. RQ: relative quantification.

After filtering the gene expression data with the significance of correlation, the level of expression, and the coefficient of variation, there were 39 and 15 genes that showed significant negative and positive correlation with SED, respectively (Table 2). Among the significantly correlated genes with highest p value, there were genes associated with extracellular matrix remodeling such as ADAMTS12 and MMP13 (Fig 5a). MMP13 was significantly associated with SED in both ER positive and ER negative subsets while ADAMTS12 showed marginal significance in ER negative tumors (Fig 5b). We tested the association between the MMP13 expression and SED in an independent cohort of 52 breast cancer patients by qRT-PCR using fresh tissues obtained from the curative surgeries. As shown in the Fig 5c, the MMP13 expression was significantly associated with the SED of the 52 tumors, validating our initial observations. These results suggest that tumor-stromal interaction mediated by MMP13 can contribute to the spatial tumor shape development in breast cancers.

**Table 2:** List of differentially expressed genes according to the SED.

Gene Symbol	Gene Name	Correlation	p-Value	Means	SD^1^	CV^2^
**ADAMTS12**	ADAM Metallopeptidase With Thrombospondin Type 1 Motif, 12	-0.536	4.36E-04	277.87	278.05	1.00
**POU2F3**	POU Class 2 Homeobox 3	-0.533	4.75E-04	133.21	158.64	1.19
**FAIM2**	Fas Apoptotic Inhibitory Molecule 2	-0.512	8.71E-04	91.08	149.86	1.65
**RFFL**	Ring Finger And FYVE-Like Domain Containing E3 Ubiquitin Protein Ligase	-0.507	9.86E-04	107.06	107.20	1.00
**C9orf172**	Chromosome 9 Open Reading Frame 172	-0.498	1.24E-03	33.73	36.55	1.08
**MMP13**	Matrix Metallopeptidase 13	-0.476	2.18E-03	1363.90	2244.65	1.65
**PDE10A**	Phosphodiesterase 10A	-0.474	2.28E-03	274.26	378.61	1.38
**GRIN1**	Glutamate Receptor, Ionotropic, N-Methyl D-Aspartate 1	-0.463	3.03E-03	93.51	148.72	1.59
**PPP1R1A**	Protein Phosphatase 1, Regulatory (Inhibitor) Subunit 1A	-0.461	3.12E-03	145.01	237.42	1.64
**WNT7B**	Wingless-Type MMTV Integration Site Family, Member 7B	-0.460	3.23E-03	248.82	280.13	1.13
**FAM171A2**	Family With Sequence Similarity 171, Member A2	-0.455	3.59E-03	58.59	88.96	1.52
**S100A7A**	S100 calcium binding protein A7A	-0.451	4.00E-03	369.17	1262.58	3.42
**SCUBE3**	signal peptide, CUB domain, EGF-like 3	-0.446	4.39E-03	517.04	794.36	1.54
**LRRC26**	leucine rich repeat containing 26	-0.445	4.55E-03	208.69	479.11	2.30
**LOC100508781**	-	-0.444	4.60E-03	37.94	45.43	1.20
**NKD1**	naked cuticle homolog 1 (Drosophila)	-0.443	4.71E-03	90.43	103.83	1.15
**KMO**	kynurenine 3-monooxygenase (kynurenine 3-hydroxylase)	-0.441	4.91E-03	497.23	653.93	1.32
**PPM1L**	protein phosphatase, Mg2+/Mn2+ dependent, 1L	-0.440	5.12E-03	92.01	118.89	1.29
**GYG2**	glycogenin 2	-0.439	5.15E-03	218.81	227.21	1.04
**PRRT4**	proline-rich transmembrane protein 4	-0.439	5.16E-03	21.88	41.19	1.88
**MNX1**	motor neuron and pancreas homeobox 1	-0.439	5.22E-03	44.43	52.79	1.19
**PCDHGB1**	protocadherin gamma subfamily B, 1	-0.433	5.85E-03	67.47	105.60	1.57
**SSH2**	slingshot protein phosphatase 2	-0.433	5.92E-03	762.60	766.67	1.01
**NPTX2**	neuronal pentraxin II	-0.432	5.99E-03	62.32	98.66	1.58
**S100A7**	S100 calcium binding protein A7	-0.432	6.05E-03	1222.79	3132.80	2.56
**LRTM2**	leucine-rich repeats and transmembrane domains 2	-0.430	6.27E-03	26.35	77.89	2.96
**STS**	steroid sulfatase (microsomal), isozyme S	-0.429	6.43E-03	995.71	2133.90	2.14
**LOC728763**	PREDICTED: Homo sapiens rootletin-like (LOC728763)	-0.429	6.43E-03	48.41	87.09	1.80
**LRRC4**	leucine rich repeat containing 4	-0.427	6.65E-03	83.92	122.05	1.45
**GUCY1A2**	guanylate cyclase 1, soluble, alpha 2	-0.424	7.22E-03	540.97	583.50	1.08
**PLCH1**	phospholipase C, eta 1	-0.422	7.38E-03	475.02	522.92	1.10
**SIGLEC15**	sialic acid binding Ig-like lectin 15	-0.421	7.66E-03	32.75	46.48	1.42
**ARSH**	arylsulfatase family, member H	-0.421	7.69E-03	31.82	69.13	2.17
**SYT12**	synaptotagmin XII	-0.420	7.78E-03	835.06	1346.86	1.61
**ERBB2**	erb-b2 receptor tyrosine kinase 2	-0.419	8.00E-03	37299.57	74157.39	1.99
**KALRN**	kalirin, RhoGEF kinase	-0.418	8.02E-03	435.61	644.51	1.48
**EPGN**	epithelial mitogen	-0.413	9.02E-03	34.51	57.23	1.66
**UNC5A**	unc-5 netrin receptor A	-0.412	9.13E-03	137.24	248.01	1.81
**SH2B2**	SH2B adaptor protein 2	-0.411	9.43E-03	58.66	60.36	1.03
**LOC101929385**	-	0.409	9.66E-03	37.76	41.64	1.10
**PTGDS**	prostaglandin D2 synthase 21kDa (brain)	0.410	9.58E-03	249.54	385.03	1.54
**DOC2A**	double C2-like domains, alpha	0.410	9.57E-03	105.05	106.28	1.01
**LOC101929724**	-	0.411	9.31E-03	13.16	17.31	1.32
**FBXO2**	F-box protein 2	0.414	8.86E-03	69.33	71.74	1.03
**HSPB6**	heat shock protein, alpha-crystallin-related, B6	0.421	7.54E-03	76.28	92.89	1.22
**KRT14**	keratin 14, type I	0.430	6.25E-03	1546.92	2735.67	1.77
**FLJ35934**	Homo sapiens FLJ35934 (FLJ35934), long non-coding RNA	0.433	5.84E-03	11.96	13.74	1.15
**LOC100506834**	Homo sapiens uncharacterized LOC100506834 (LOC100506834), long non-coding RNA	0.435	5.69E-03	32.52	33.07	1.02
**LOC102723354**	Homo sapiens uncharacterized LOC102723354 (LOC102723354), long non-coding RNA	0.436	5.49E-03	19.35	22.70	1.17
**MIR143HG**	MIR143 host gene	0.439	5.18E-03	28.06	36.70	1.31
**MIR145**	microRNA 145	0.447	4.29E-03	11.98	21.78	1.82
**ACKR1**	atypical chemokine receptor 1 (Duffy blood group)	0.450	4.03E-03	165.78	260.50	1.57
**MIR29C**	microRNA 29c	0.461	3.12E-03	47.22	52.91	1.12
**LOC101930481**	-	0.471	2.48E-03	11.40	19.03	1.67

^1^: Standard deviation,

^2^: Coefficient of variation

**Fig 6:**
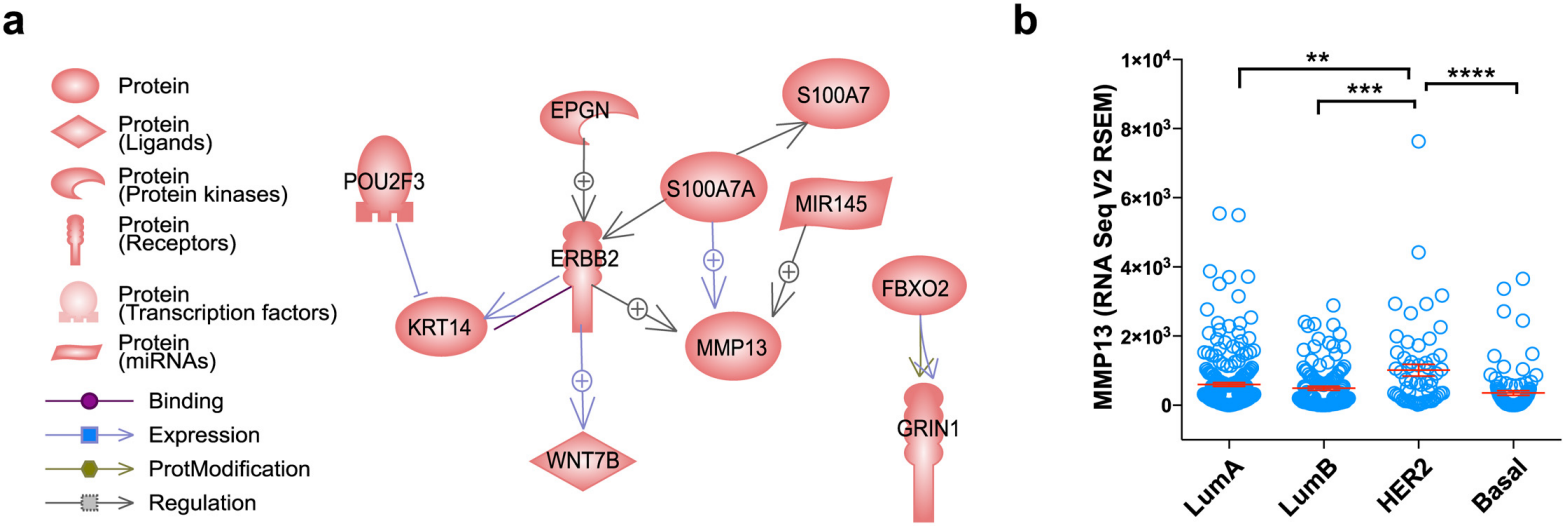
Molecular regulatory network for MMP13 and its expression in various subtypes of breast cancers. The interaction network analysis showing a potential regulatory pathway of MMP13 based on the Pathway Studio Web (a), and the levels of MMP13 expression in TCGA dataset according to the PAM50 molecular subtypes (b) are shown.

Molecular network analysis of the genes having correlated expression with SED is shown in Fig 6a. Network analysis shows that MMP13 gene expression can be positively regulated by ERBB2 and S100A7A, both of which are negatively correlated with SED. The relationship between MMP13 and ERBB2 activity was further investigated by using TCGA RNA Seq dataset. The expression level of MMP13 was significantly higher in HER2-enriched subtype when compared to those of other molecular subtypes (Fig 6b).

The degree of cancer cell clonality and the degree of global somatic mutation were also tested for their association with the tumor eccentricity. The numbers of cancer cell clonality and the numbers of the somatic mutations for each tumor were examined by using the whole exome sequencing data of the same 39 tumors. Neither the cancer cell clonality nor the degree of somatic mutation showed significant correlation with the SED (S1 Fig).

## Discussion

In this study, we aimed to describe the clinical relevance of the three-dimensional growth patterns in breast cancer and the biologic explanation underlying the diverse tumor growth patterns. First, we have demonstrated that the ellipsoid tumor volume measurement, which is theoretically more accurate way of estimating true tumor volume, can result in substantial changes in tumor size determination. Wapnir et al [3] has also demonstrated that there is a significant overestimation of tumor volume when using the greatest diameter alone for early breast cancers. They have also suggested that by measuring tumor volume, one can improve the accuracy of prognosis prediction in early breast cancer in their analysis of 165 breast cancer patients. However, our results show that classifying patients according to their tumor volumes did not improve the outcome prediction compared to the current tumor size measurement. The implication of this finding is that measuring the total amount of cancer cell does not provide additional information on the tumor aggressiveness to the conventional measurements. Our finding support the idea that a small proportion of cancer cells in solid tumors, mostly located at the invading front, determines the local invasion and metastasis while the remaining tumor cells are non-metastastic [16]. Also, it is consistent with our previous report showing that the presence of the additional invasive tumors does not lead to worse outcomes in luminal subtypes of breast cancer [17].

Our results also show that the molecular subtypes of breast cancer affect the patterns of the 3-dimensional tumor growth in breast tumors. Three-dimensional tumor diameters, measured by pathologic examination, showed that triple negative tumors had least eccentric shape when compared to other subtypes. Recent breast imaging studies have also shown similar findings showing triple negative tumors having more round shape and smooth margins [18]. In consistent with our results, Bae et al [19] have shown by quantitative MR imaging of 280 breast cancer patients that triple negative tumors have more round tumor shape than other major subtypes. In addition to its association with molecular subtype, the spatial tumor growth pattern in breast cancer, measured by SED, is also associated with known prognostic features such as tumor size, lymph node metastasis, and histologic grade. The tumors with high eccentricity (high SED) showed worse survival outcome due to the high incidences of the unfavorable prognostic factors.

Our transcriptome analysis data of human breast cancer tissues suggests a potential link between the spatial growth patterns and the expression levels of extracellular matrix remodeling genes such as MMP13 which show a significant negative correlation with the SED. Network analysis showed that MMP13 gene expression can be regulated by ERBB2 and S100A7A, both of which also showed negative correlation with SED. ERBB2 is a major gene that determine the molecular subtype of breast cancer [20] and S100A7A has been shown to be down-regulated in estrogen receptor negative tumors [21]. Furthermore, our analysis of TCGA data further showed that MMP13 expression is significantly higher in HER2-enriched subtype and triple negative subtype showed lowest MMP13 expression. Our observation can generate a hypothesis that ERBB2 and S100A7A-mediated MMP13 expression can contribute to the subtype-specific spatial growth patterns in breast cancer.

On the other hand, our study cannot fully explain the molecular mechanisms underlying the relationship between the increased eccentricity and higher incidence of poor prognostic factors in breast cancer. Some of the genes having negative correlation with SED were reported to accelerate breast cancer progression. For example, previous studies have shown pro-tumorigenic and pro-metastatic role of ERBB2, WNT7b, S100A7, and MMP13 in breast cancer [22–27]. However, Visozo et al [28] have shown that the protein expression levels of MMP13 showed negative correlation with advanced tumor stages suggesting a potential tumor-suppressive role of MMP13 in breast cancers. Furthermore, a recent study showed that silencing MMP13 in the stromal cells increased the rate of mammary cancer metastasis via mechanisms involving peri-tumoral collagen I remodeling [29]. These findings suggest a complex role of MMP13 within the tumor microenvionment, which needs further exploration.

Our study carries several important limitations. First, our assessment of tumor shape is based on the pathologically measured tumor diameters. The complex contour of the human breast cancer cannot be fully reflected by the dimensional diameter alone. An improved tumor shape assessment such as an automated imaging tool may give a better understanding into the spatial tumor shape formation. Second, our RNA sequencing data was derived from a small number of patients. Expanding the analysis to larger patient cohort for whom both transcriptome data and spatial shape data are available can give more clear insight into the molecular mechanism underlying the tumor shape heterogeneity.

## Conclusion

In conclusion, our present study demonstrates the accuracy of current tumor size measurement system in predicting breast cancer survival. However, during the analysis, we have observed a significant difference between breast cancer molecular subtypes in terms of tumor eccentricity. The degree of eccentricity was associated with various known prognostic factors such as tumor size and lymph node metastasis. Additionally, by using RNA sequencing, we show that the genes involved in the extracellular matrix remodeling such as MMP13 may contribute to the tumor shape heterogeneity.

## Supporting Information

S1 Fig The relationship between the tumor eccentricity and the cancer cell clonality and the degree of somatic mutations. The correlation between the numbers of total somatic mutations in tumor tissue (a) and the numbers of cancer cell clonality (b) against the spheroid-ellipsoid discrepancy (SED).
(EPS)

S1 Table The multivariate Cox regression analysis on distant-metastasis free survival. (XLSX)
